# Complete Genome Sequence of a *Fusarium graminearum* Double-Stranded RNA Virus in a Newly Proposed Family, *Alternaviridae*

**DOI:** 10.1128/genomeA.00064-18

**Published:** 2018-02-22

**Authors:** Hao He, Xiaoguang Chen, Pengfei Li, Dewen Qiu, Lihua Guo

**Affiliations:** a State Key Laboratory for Biology of Plant Diseases and Insect Pests, Institute of Plant Protection, Chinese Academy of Agricultural Sciences, Beijing, China

## Abstract

We describe here a double-stranded RNA mycovirus, termed *Fusarium graminearum alternavirus 1* (FgAV1/AH11), from the isolate AH11 of the phytopathogenic fungus *F. graminearum*. Phylogenetic analysis showed that FgAV1/AH11 belongs to a newly proposed family, *Alternaviridae*. This is the first report of a mycovirus in the family *Alternaviridae* that infects *F. graminearum*.

## GENOME ANNOUNCEMENT

Mycoviruses with double-stranded RNA (dsRNA) genomes are grouped into seven families (*Reoviridae*, *Partitiviridae*, *Chrysoviridae*, *Totiviridae*, *Quadriviridae*, *Megabirnaviridae*, and *Endornaviridae*) and one genus (*Botybirnavirus*) (1). Hammond et al. (2) proposed to establish a new genus, *Alternavirus*, and a novel family, *Alternaviridae*, to accommodate *Alternaria alternata virus 1* (AaV1) and *Aspergillus mycovirus 341* (AMV). To date, only four reported mycoviruses belong to the family *Alternaviridae*, namely, AMV (1), AaV1 (3), *Aspergillus foetidus mycovirus* (AfMV) (4), and *Fusarium poae alternavirus 1* (FpAV1) (5).

The AH11 strain of *F. graminearum* was incubated in potato dextrose broth for 4 days at 25°C and 180 rpm in the dark. The mycelia collected were used for dsRNA extraction by the CF-11 cellulose chromatography method. Random primers (5′-GACGTCCAGATCGCGAATTCNNNNNN-3′) were used to synthesize cDNAs (TransGen) from purified dsRNA. The resulting cDNAs were amplified using one specific primer (5′-GACGTCCAGATCGCGAATTC-3′) and 2× TransTaq high-fidelity PCR super mix (TransGen). The amplified PCR products were purified, ligated to the PMD18-T vectors, and transformed into Trans T1 chemically competent cells (TransGen) for sequencing. Based on the sequences obtained, dsRNA-specific primers were designed for reverse transcription PCR. The 3′ RNA ligase-mediated rapid amplification of cDNA ends (RLM RACE) protocol was performed as described previously to clone the termini of the dsRNAs (6, 7).

Agarose gel electrophoresis and sequence analysis of the AH11 strain dsRNA extraction indicated the presence of the segments dsRNA1, dsRNA2, and dsRNA3. The complete nucleotide sequences of dsRNA1, dsRNA2, and dsRNA3, excluding the poly(A) tails at their 3' termini, were 3,524 bp, 2,470 bp, and 2,460 bp in length, respectively. FgAV1/AH11 dsRNA1, dsRNA2, and dsRNA3 each contained a single putative open reading frame (ORF1, ORF2, and ORF3, respectively). ORF1 (nucleotide [nt] positions 81 to 3452) of FgAV1/AH11 dsRNA1 was found to encode a putative protein of 1,123 amino acids (aa) with a predicted molecular mass of 126 kDa and contained a conserved domain corresponding to RNA-dependent RNA polymerase (RdRp) (nt positions 1245 to 2336). However, no conserved domains were detected in the putative proteins encoded by ORF2 (756 aa, 83.5 kDa) and ORF3 (743 aa, 81.1 kDa). To study the relatedness between FgAV1/AH11 and other dsRNA viruses, a phylogenetic tree based on the putative RdRp sequence of ORF1 revealed that FgAV1/AH11 formed an independent phylogenetic branch together with FgAV1/AH11, AaV1, AfMV, and AMV. Among these four viruses, FgAV1/AH11 was most closely related to FpAV1 with 98% (ORF1), 99% (ORF2), and 98% (ORF3) aa sequence identities, suggesting that FgAV1/AH11 and FpAV1 belong to the same virus species. However, the genomic composition of this family differed between AaV1 (4 segments, 1.4 to 3.6 kbp) (3), AfMV (4 segments, 2.0 to 3.6 kbp) (4), AMV (4 segments, 1.5 to 3.6 kbp) (2), and FpAV1 (3 segments, 2.4 to 3.6 kbp) (5). FgAV1/AH11 and FpAV1 both lack the fourth segment. Thus, we concluded that FgAV1/AH11 belonged to the newly proposed family *Alternaviridae*. This is the first report of a dsRNA mycovirus in the family *Alternaviridae* that infects *F. graminearum*.

### Accession number(s).

The whole-genome sequence of FgAV1/AH11 has been deposited in GenBank under the accession no. MG254901, MG254902, and MG697236.

## References

[1] Kotta-Loizou I, Coutts RHA 2017 Mycoviruses in *Aspergilli*: a comprehensive review. Front Microbiol 8:1699. doi:10.3389/fmicb.2017.01699. 28932216 PMC5592211

[2] Hammond TM, Andrewski MD, Roossinck MJ, Keller NP 2008 *Aspergillus* mycoviruses are targets and suppressors of RNA silencing. Eukaryot Cell 7:350–357. doi:10.1128/EC.00356-07. 18065651 PMC2238147

[3] Aoki N, Moriyama H, Kodama M, Arie T, Teraoka T, Fukuhara T 2009 A novel mycovirus associated with four double-stranded RNAs affects host fungal growth in *Alternaria alternata*. Virus Res 140:179–187. doi:10.1016/j.virusres.2008.12.003. 19118588

[4] Kozlakidis Z, Herrero N, Ozkan S, Kanhayuwa L, Jamal A, Bhatti MF, Coutts RHA 2013 Sequence determination of a quadripartite dsRNA virus isolated from *Aspergillus foetidus*. Arch Virol 158:267–272. doi:10.1007/s00705-012-1362-3. 22760661

[5] Osaki H, Sasaki A, Nomiyama K, Tomioka K 2016 Multiple virus infection in a single strain of *Fusarium poae* shown by deep sequencing. Virus Genes 52:835–847. doi:10.1007/s11262-016-1379-x. 27550368

[6] Chiba S, Salaipeth L, Lin YH, Sasaki A, Kanematsu S, Suzuki N 2009 A novel bipartite double-stranded RNA mycovirus from the white root rot fungus *Rosellinia necatrix*: molecular and biological characterization, taxonomic considerations, and potential for biological control. J Virol 83:12801–12812. doi:10.1128/JVI.01830-09. 19828620 PMC2786845

[7] Xie J, Wei D, Jiang D, Fu Y, Li G, Ghabrial S, Peng Y 2006 Characterization of debilitation-associated mycovirus infecting the plant-pathogenic fungus *Sclerotinia sclerotiorum*. J Gen Virol 87:241–249. doi:10.1099/vir.0.81522-0. 16361437

